# A review of ferric citrate clinical studies, and the rationale and design of the Ferric Citrate and Chronic Kidney Disease in Children (FIT4KiD) trial

**DOI:** 10.1007/s00467-022-05492-7

**Published:** 2022-03-02

**Authors:** Mark R. Hanudel, Marciana L. Laster, Anthony A. Portale, Aditi Dokras, Raymond P. Quigley, German A. Lozano Guzman, Joshua J. Zaritsky, Nicole A. Hayde, Frederick J. Kaskel, Mark M. Mitsnefes, Jorge A. Ramirez, Peace D. Imani, Poyyapakkam R. Srivaths, Amy J. Kogon, Michelle R. Denburg, Tom D. Blydt-Hansen, Loretta Z. Reyes, Larry A. Greenbaum, Darcy K. Weidemann, Bradley A. Warady, David A. Elashoff, Susan R. Mendley, Tamara Isakova, Isidro B. Salusky

**Affiliations:** 1 Department of Pediatrics, Division of Nephrology, David Geffen School of Medicine at UCLA and UCLA Mattel Children’s Hospital, 10833 Le Conte Avenue, Los Angeles, CA 90095 USA; 2 Department of Pediatrics, Division of Nephrology, UCSF School of Medicine and UCSF Benioff Children’s Hospital, San Francisco, CA USA; 3 Department of Pediatrics, Division of Nephrology, Southwestern School of Medicine and Children’s Medical Center, University of Texas, Dallas, TX USA; 4 Department of Pediatrics, Division of Nephrology, Tower Health and St. Christopher’s Hospital for Children, Philadelphia, PA USA; 5 Department of Pediatrics, Division of Nephrology, Albert Einstein College of Medicine and Children’s Hospital at Montefiore, Bronx, NY USA; 6 Department of Pediatrics, Division of Nephrology, Cincinnati Children’s Hospital Medical Center, Cincinnati, OH USA; 7 Department of Pediatrics, Division of Nephrology, Arnold Palmer Hospital for Children, Orlando, FL USA; 8 Department of Pediatrics, Division of Nephrology, Baylor College of Medicine and Texas Children’s Hospital, Houston, TX USA; 9 Department of Pediatrics, Division of Nephrology, Perelman School of Medicine at the University of Pennsylvania and Children’s Hospital of Philadelphia, Philadelphia, PA USA; 10 Department of Pediatrics, Division of Nephrology, University of British Columbia and British Columbia Children’s Hospital, Vancouver, BC Canada; 11 Department of Pediatrics, Division of Nephrology, Emory University School of Medicine and Children’s Healthcare of Atlanta, Atlanta, GA USA; 12 Department of Pediatrics, Division of Nephrology, University of Missouri-Kansas City School of Medicine and Children’s Mercy Kansas City, Kansas City, MO USA; 13 Department of Medicine/Biostatistics, Division of General Internal Medicine and Health Service Research, David Geffen School of Medicine at UCLA, Los Angeles, USA; 14 National Institute of Diabetes and Digestive and Kidney Diseases, Division of Kidney, Urologic, and Hematologic Diseases, National Institutes of Health, Bethesda, MD USA; 15 Department of Medicine, Division of Nephrology, Northwestern University Feinberg School of Medicine, Chicago, IL USA

**Keywords:** Pediatrics, Chronic kidney disease, Ferric citrate, Fibroblast growth factor 23

## Abstract

Pediatric chronic kidney disease (CKD) is characterized by many co-morbidities, including impaired growth and development, CKD-mineral and bone disorder, anemia, dysregulated iron metabolism, and cardiovascular disease. In pediatric CKD cohorts, higher circulating concentrations of fibroblast growth factor 23 (FGF23) are associated with some of these adverse clinical outcomes, including CKD progression and left ventricular hypertrophy. It is hypothesized that lowering FGF23 levels will reduce the risk of these events and improve clinical outcomes. Reducing FGF23 levels in CKD may be accomplished by targeting two key stimuli of FGF23 production—dietary phosphate absorption and iron deficiency. Ferric citrate is approved for use as an enteral phosphate binder and iron replacement product in adults with CKD. Clinical trials in adult CKD cohorts have also demonstrated that ferric citrate decreases circulating FGF23 concentrations. This review outlines the possible deleterious effects of excess FGF23 in CKD, summarizes data from the adult CKD clinical trials of ferric citrate, and presents the Ferric Citrate and Chronic Kidney Disease in Children (FIT4KiD) study, a randomized, placebo-controlled trial to evaluate the effects of ferric citrate on FGF23 in pediatric patients with CKD stages 3–4 (ClinicalTrials.gov Identifier NCT04741646).

**Supplementary Information:**

The online version contains supplementary material available at 10.1007/s00467-022-05492-7.

## Introduction

Childhood and adolescence are crucial times for healthy growth and development. Children with chronic kidney disease (CKD) suffer from suboptimal growth, impaired neurocognitive development, and multisystemic organ dysfunction, which manifests as multiple CKD-associated co-morbidities, including CKD-mineral bone disorder (CKD-MBD) [1, 2], dysregulated iron metabolism [3, 4], anemia [3], and cardiovascular disease [5]. The adverse effects of these CKD-associated co-morbidities can have long-lasting consequences that persist into adulthood, contributing to a markedly reduced life expectancy for children with CKD [6]. Amelioration of these conditions may improve clinical outcomes for pediatric patients with CKD.

Contributing to these interrelated CKD co-morbidities is fibroblast growth factor 23 (FGF23), a predominantly bone-derived phosphaturic hormone. Circulating concentrations of FGF23 increase early in the course of adult [7] and pediatric [8] CKD—before other traditional markers of CKD-MBD such as serum phosphate and parathyroid hormone—and continue to increase as kidney function declines. In both adult and pediatric CKD cohorts, higher FGF23 concentrations are independently associated with adverse clinical outcomes, including left ventricular hypertrophy [9, 10] and CKD progression [11–13]. Multiple factors stimulate FGF23 production, including dietary phosphate absorption [14, 15] and iron deficiency [16]. Targeting the stimuli of FGF23 production may lower circulating FGF23 concentrations and may reduce the development of FGF23-associated adverse clinical outcomes.

**Fig. 1:**
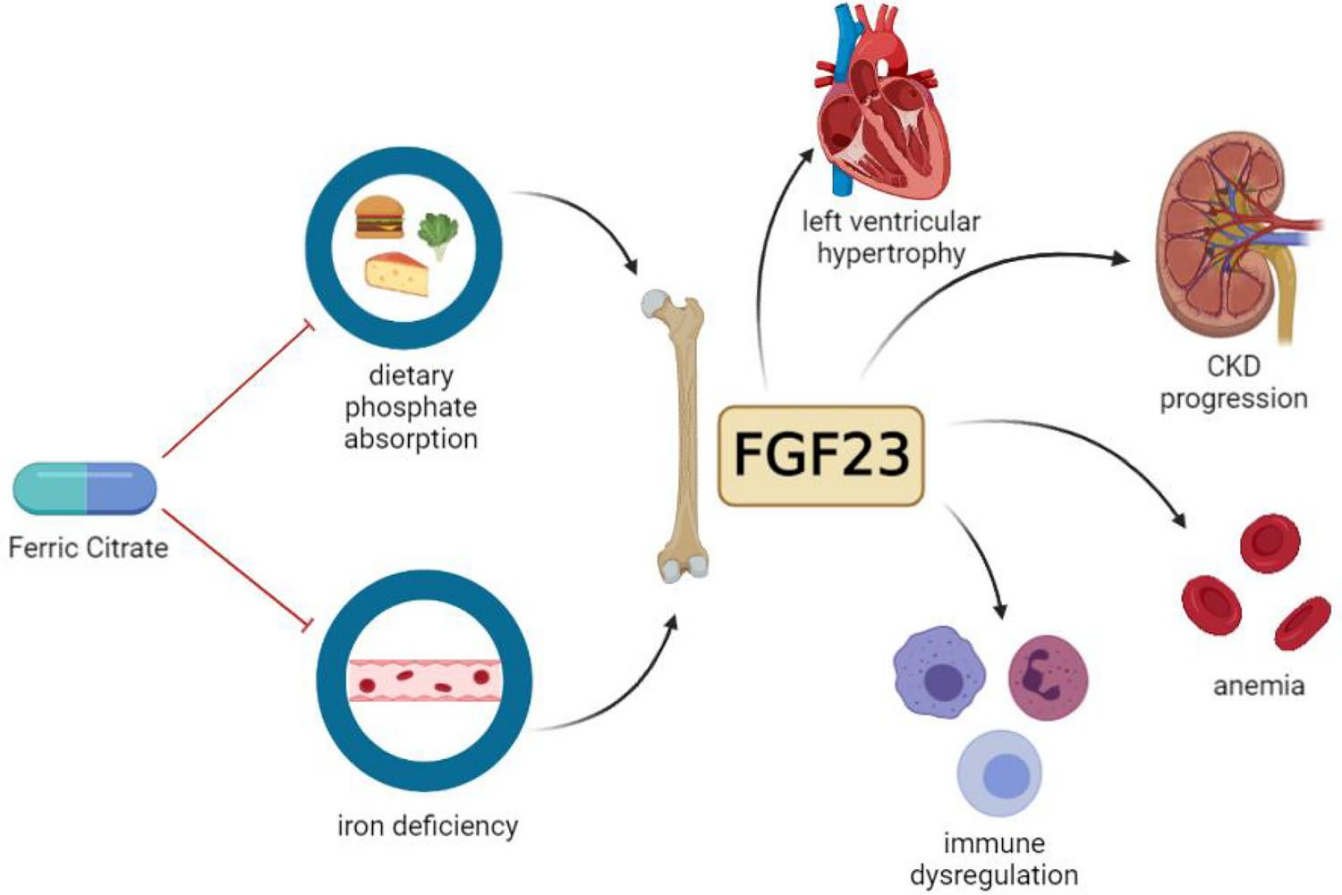
Ferric citrate inhibits two stimuli of FGF23 production, dietary phosphate absorption and iron deficiency. Lowering circulating FGF23 concentrations may prevent the development of various FGF23-associated, off-target, adverse effects

Recently, ferric citrate (Auryxia, Akebia Therapeutics, Inc., Cambridge, MA) was approved for clinical use as an enteral phosphate binder in adult patients with CKD on dialysis, and as an iron replacement product in adult patients with non-dialysis-dependent CKD and iron deficiency anemia [17]. By decreasing dietary phosphate absorption and improving iron status, ferric citrate may mitigate two stimuli for excess FGF23 production in CKD, leading to decreased circulating FGF23 concentrations (Fig. 1). In this review, we will describe the potentially deleterious effects of FGF23 in CKD; summarize the published data from clinical trials in adults evaluating ferric citrate in CKD; and present the background, hypotheses, and design features of the NIDDK-funded Ferric Citrate and Chronic Kidney Disease in Children (FIT4KiD) study, a 12-month, double-blind, randomized, placebo-controlled trial to evaluate the effects of ferric citrate on changes in FGF23 levels in pediatric patients with CKD stages 3–4.

### Fibroblast growth factor 23

FGF23 is an essential hormone secreted mainly by osteocytes that physiologically regulates phosphate and 1,25-dihydroxyvitamin D (1,25(OH)_2_D). FGF23 decreases expression of the type II sodium-phosphate cotransporters (NaPi-2a and NaPi-2c) in renal proximal tubules, reducing renal phosphate reabsorption [18, 19]. FGF23 also decreases expression of renal 1α-hydroxylase, the enzyme that converts 25(OH)D to active 1,25(OH)_2_D, and increases expression of renal 24-hydroxylase, the enzyme that converts 25(OH)D and 1,25(OH)_2_D to inactive metabolites, thus decreasing overall renal 1,25(OH)_2_D production [20–22]. Decreased renal phosphate reabsorption and decreased 1,25(OH)_2_D-mediated enteral phosphate absorption results in decreased serum phosphate concentrations. Therefore, in the setting of phosphate loading or hyperphosphatemia, an increase in circulating FGF23 levels directly induces phosphate excretion and indirectly reduces dietary phosphate absorption, thus maintaining normal circulating phosphate concentrations. In the setting of CKD, bone [23] and circulating [7, 8, 24, 25] levels of FGF23 increase early and continue to increase as kidney function declines [7, 8, 24–26], helping to maintain normophosphatemia until late-stage CKD [7, 8].

### Adverse effects of increased FGF23 concentrations in CKD

Although progressively increasing FGF23 concentrations in CKD help to prevent or mitigate hyperphosphatemia, elevated FGF23 levels have also been independently associated with a multitude of adverse “off-target” effects. Most notably, FGF23 has emerged as a potential mediator of cardiac hypertrophy, independent of hypertension and vascular calcification [27]. Studies conducted in vitro and in mice have demonstrated that FGF23 directly induces cardiac myocyte hypertrophy [9] by binding to cardiomyocyte FGF receptor 4 (FGFR4), inducing the downstream phosphorylation of phospholipase Cγ (PLCγ) and the activation of calcineurin-nuclear factor of activated T-cells (NFAT) signaling pathways that affect genes regulating cardiac remodeling [28, 29]. Consistent with these pre-clinical observations, in both the adult Chronic Renal Insufficiency Cohort (CRIC) study [9] and the pediatric Chronic Kidney Disease in Children (CKiD) study [10], higher circulating FGF23 concentrations were independently associated with left ventricular hypertrophy. Furthermore, FGF23 may have additional adverse cardiac effects. Pre-clinical studies demonstrate that FGF23 alters cardiomyocyte intracellular calcium concentrations [30, 31] and induces pro-arrhythmogenic activity [31]. Potentially consistent with these observations, in the CRIC study, higher FGF23 levels were independently associated with prevalent and incident atrial fibrillation [32].

Increased circulating FGF23 concentrations in CKD may also have extra-cardiac adverse effects. In multiple adult (CRIC [12] and the Mild to Moderate Kidney Disease (MMKD) study [11]) and pediatric (CKiD [13]) cohorts, higher FGF23 levels were independently associated with a more rapid rate of CKD progression, even after adjustment for traditional CKD progression risk factors. Regarding CKD-MBD, FGF23-mediated suppression of 1,25(OH)_2_D production promotes secondary hyperparathyroidism [33]. Regarding CKD-associated anemia, murine in vivo studies suggest that FGF23 may have inhibitory effects on erythropoiesis [34, 35] and, in the CRIC study, higher FGF23 levels were independently associated with prevalent and incident anemia [36]. FGF23 may also impact the immune system, as pre-clinical studies demonstrate that in CKD, increased FGF23 levels are associated with impaired neutrophil activation [37]. In the Hemodialysis (HEMO) study cohort, higher FGF23 levels were independently associated with infection-related hospitalization or death [38]. Possibly due to these cardiac and extra-cardiac adverse effects, higher circulating concentrations of FGF23 in CKD are independently associated with increased overall mortality rates [12, 39, 40].

### FGF23 production

Given the possible multisystemic adverse effects of increased FGF23 concentrations in CKD, it can be hypothesized that lowering FGF23 levels in patients with CKD may improve clinical outcomes. One potential mechanism to lower FGF23 levels is to target the stimuli that increase FGF23 production. Multiple factors stimulate FGF23 production, central among which is dietary phosphate absorption [14, 15]. In studies of healthy volunteers, a phosphate-depleted diet decreased circulating FGF23 concentrations [14, 15], and a phosphate-loaded diet increased circulating FGF23 concentrations [15].

Recently, iron deficiency has been identified as a novel stimulus of FGF23 production. Several murine studies demonstrate that iron deficiency potently induces *Fgf23* mRNA expression [41–44]. Iron deficiency also concurrently increases intracellular FGF23 post-translational proteolytic cleavage, resulting in secretion of FGF23 protein fragments from the cell [41–44]. However, in CKD, FGF23 proteolytic cleavage may be impaired [45–47], thus uncoupling iron deficiency-induced increased FGF23 transcription from its post-translational cleavage. Therefore, in the *absence* of CKD, iron deficiency results in increased circulating concentrations of FGF23 fragments; however, in the *presence* of CKD, iron deficiency may increase concentrations of full-length FGF23. Indeed, in a pre-clinical study of mice with and without experimental CKD fed an iron-deficient diet, whole bone *Fgf23* mRNA expression and plasma concentrations of total (intact + fragmented) FGF23 increased to a similar degree in both the non-CKD and CKD groups, but the increase in plasma concentrations of full-length, intact FGF23 was much greater in the mice with CKD [44]. The median percentage of circulating FGF23 that was intact was only 12% in the non-CKD iron-deficient mice, but was 72% in the CKD iron-deficient group [44]. Therefore, iron deficiency, which is common in pediatric patients with CKD [48, 49], may represent a novel factor contributing to elevated intact FGF23 levels in CKD.

For human samples, there are commercial assays to measure both C-terminal FGF23 and intact FGF23 [50]. The C-terminal assay captures both intact FGF23 and C-terminal FGF23 fragments, thus measuring total (intact + fragmented) FGF23 concentrations. Conversely, the intact assay captures only intact FGF23 (iFGF23). In human CKD cohorts, iron deficiency is associated with increased concentrations of circulating total FGF23, as measured by the C-terminal FGF23 assay [51, 52]. Associations between iron status and intact FGF23 levels have not been specifically evaluated in large CKD cohorts; however, given that FGF23 cleavage is impaired in CKD [45–47], it is hypothesized that iron deficiency in CKD would increase intact FGF23 to a greater degree than iron deficiency in the absence of CKD would. In the FIT4KiD trial, the primary outcome is changes in iFGF23, as this is considered to be the biologically active FGF23 moiety, but total FGF23 concentrations will also be measured as an exploratory analysis.

### Randomized controlled trials of ferric citrate in adult patients with non-dialysis-dependent CKD

As ferric citrate can function as both a phosphate binder and a source of iron, it can both decrease enteral phosphate absorption and improve iron status in CKD, thus targeting two of the mechanisms that increase FGF23 levels. Multiple randomized, placebo-controlled trials have evaluated the effects of ferric citrate on serum phosphate, iron status, and FGF23 levels in adult patients with non-dialysis-dependent CKD. In a study published in 2014, Yokoyama et al. conducted a 12-week, randomized trial of ferric citrate vs. placebo in 86 Japanese patients with CKD stages 3–5 [53]. Compared to placebo, ferric citrate decreased serum phosphate concentrations, increased transferrin saturation and ferritin, and decreased circulating iFGF23 levels. In a similar study published in 2015, Block et al. conducted a 12-week, randomized trial of ferric citrate vs. placebo in 141 American patients with CKD stages 3–5 [54]. Compared to placebo, ferric citrate decreased serum phosphate concentrations; increased transferrin saturation, ferritin, and hemoglobin; and decreased circulating iFGF23 levels. Lastly, in a study published in 2017, Fishbane et al. conducted a 16-week, randomized trial of ferric citrate vs. placebo in 233 American patients with CKD stages 3–5 [55]. Compared to placebo, ferric citrate decreased serum phosphate concentrations; increased transferrin saturation, ferritin, and hemoglobin; and decreased circulating iFGF23 levels. More recently, in a study published in 2019, Block et al. conducted a 36-week randomized trial of ferric citrate vs. standard of care in 199 patients with advanced CKD (eGFR < 20 ml/min/1.73 m^2^) [56]. Compared to standard of care, ferric citrate decreased serum phosphate concentrations; increased transferrin saturation, ferritin, and hemoglobin; and decreased circulating iFGF23 levels. Details of these randomized trials are presented in Table 1, demonstrating the beneficial effects of ferric citrate on phosphate, iron, and FGF23 in the setting of CKD. The primary objective of the FIT4KiD trial, described below, is to evaluate the effects of ferric citrate on changes in circulating iFGF23 concentrations in a cohort of pediatric patients with CKD.

**Table 1:** Randomized controlled trials of ferric citrate in adult patients with non-dialysis-dependent CKD. *FC*, ferric citrate; *CKD*, chronic kidney disease; *eGFR*, estimated glomerular filtration rate; *TSAT*, transferrin saturation; *FGF23*, fibroblast growth factor 23; *LS*, least squares

Study	Study details	Study parameters	Effects of ferric citrate	*p*-value for between-group differences
Yokoyama et al. [53]	FC (*n* = 57) vs. placebo (*n* = 29) 12-week duration Inclusive of CKD stages 3–5 (mean eGFR 9 ml/min/1.73 m^2^) Mean age 65 years	Phosphate TSAT Ferritin Hemoglobin FGF23	Decreased from mean 5.7 to 4.4 mg/dl Increased from mean 27 to 44% Increased from mean 69 to 204 ng/ml Increased from mean 10.3 to 10.7 g/dl Decreased from median 453 to 209 pg/ml	< 0.001 < 0.001 < 0.001 0.23 < 0.001
Block et al. [54]	FC (*n* = 72) vs. placebo (*n* = 69) 12-week duration Inclusive of CKD stages 3–5 (mean eGFR 24 ml/min/1.73 m^2^) Mean age 65 years	Phosphate TSAT Ferritin Hemoglobin FGF23	Decreased from mean 4.5 to 3.9 mg/dl Increased from mean 22 to 32% Increased from mean 116 to 189 ng/ml Increased from mean 10.5 to 11.0 g/dl Decreased from median 159 to 105 pg/ml	< 0.001 < 0.001 < 0.001 < 0.001 0.02
Fishbane et al. [55]	FC (*n* = 117) vs. placebo (*n* = 116) 16-week duration Inclusive of CKD stages 3–5 (mean eGFR 29 ml/min/1.73 m^2^) Mean age 65 years	Phosphate TSAT Ferritin Hemoglobin FGF23	Mean relative change vs. placebo of − 0.2 mg/dl Mean relative change vs. placebo of + 18% Mean relative change vs. placebo of + 170 ng/ml Mean relative change vs. placebo of + 0.8 g/dl Decreased from median 134 to 105 pg/ml	0.02 < 0.001 < 0.001 < 0.001 < 0.001
Block et al. [56]	FC (*n* = 133) vs. usual care (*n* = 66) 36-week duration Inclusive of eGFR < 20 ml/min/1.73 m^2^ (mean eGFR 14 ml/min/1.73 m^2^) Mean age 62 years	Phosphate TSAT Ferritin Hemoglobin FGF23	Lower LS mean vs. usual care (4.2 vs. 4.6 mg/dl) Increased with FC, unchanged with usual care Increased with FC, unchanged with usual care Increased with FC, decreased with usual care Unchanged with FC, increased with usual care	< 0.001 < 0.001 < 0.001 < 0.001 < 0.001

Additionally, the 2019 trial conducted by Block et al. evaluated the effects of ferric citrate on a composite clinical endpoint that included death, provision of dialysis, or transplantation. Compared to patients randomized to standard of care, patients randomized to treatment with ferric citrate had a lower incidence of the composite endpoint [56], suggesting possible direct or indirect benefits of ferric citrate on clinical outcomes. However, in this trial, there was some imbalance in the baseline characteristics of the ferric citrate and standard of care groups, with significantly more diabetics randomized to the standard of care group. Also, this pilot study included a standard of care control, was open-label, and was conducted at a single center. Nevertheless, the encouraging results from this pilot study informed the design of a larger (1,000 patients), multicenter, randomized, double-blind, placebo-controlled trial evaluating the effects of ferric citrate on hard clinical endpoints (death, dialysis initiation, transplantation, hospitalization) in patients with advanced CKD (eGFR < 20 ml/min/1.73 m^2^) — the FRONTIER trial (Block et al. Abstract PO2381, ASN Kidney Week 2021).

### Ferric citrate use in pediatric patients with CKD

No randomized controlled trials of ferric citrate have been conducted in pediatric patients with CKD. However, one center published their real-world experience with the off-label use of ferric citrate in a small cohort of pediatric patients with kidney failure on dialysis [57]. That retrospective analysis included 11 patients on dialysis, most of whom were adolescents (median age at ferric citrate initiation 13 years, range 4 to 17 years). Ferric citrate was either added to patients’ phosphate binder regimens or patients were switched from their current phosphate binders to ferric citrate. The median duration of treatment with ferric citrate was 214 days (range 39 to 654 days). The authors compared time-averaged values for serum phosphate and iron-related parameters before and after starting ferric citrate therapy. Administration of ferric citrate decreased serum phosphate concentrations from a median (interquartile range) of 6.5 (5.5, 7.0) to 5.2 mg/dl (5.1, 6.3) (*p* = 0.014), and decreased age-adjusted phosphate standard deviation scores from 2.3 (1.5, 3.6) to 0.9 (0.0, 2.4) (*p* = 0.019). Ferric citrate therapy also increased transferrin saturation from 26 (17, 34) to 34% (28, 46) (*p* = 0.049), and increased serum ferritin from 107 (86, 675) to 230 ng/ml (113, 716) (*p* = 0.074). This retrospective study suggests that ferric citrate may be efficacious in pediatric patients with CKD, warranting further investigation in prospective clinical trials.

Ferric citrate appears to have acceptable tolerability, although close monitoring of iron parameters is warranted. In the above study, the maximum time-averaged transferrin saturation and serum ferritin observed while on ferric citrate was 55% and 1,162 ng/ml, respectively. In studies of adult patients with CKD treated with ferric citrate [54, 55], the most common adverse effects were gastrointestinal symptoms, including discolored feces, diarrhea, constipation, and nausea [58]. However, in these studies, only 5.3% of study subjects randomized to ferric citrate discontinued the study drug because of gastrointestinal adverse effects [58]. Similarly, in the above pediatric retrospective study, no patient developed gastrointestinal adverse effects severe enough to require dose reduction or discontinuation of ferric citrate. The FIT4KiD trial, described below, will assess the safety and tolerability of ferric citrate in pediatric patients with CKD.

### The FIT4KiD trial

The FIT4KiD study is a phase 2, randomized, double-blind, placebo-controlled trial, designed to evaluate the effects of ferric citrate on iFGF23 in children with CKD stages 3–4. Depending on eGFR, the prevalence of elevated FGF23 concentrations (as measured by the total FGF23 assay) in children with CKD stages 3–4 is ~ 60–100% [8]. FIT4KiD was approved and is funded by NIH/NIDDK through a cooperative agreement (U01-DK122013) among 12 clinical sites (Table 2). The study protocol was approved by a central Institutional Review Board (Washington University in St. Louis, IRB #202012083, approved 2/26/21) and by the Data and Safety Monitoring Board appointed by NIDDK (approved on 6/2/20).

**Table 2:** Ferric Citrate and Chronic Kidney Disease in Children (FIT4KiD) participating sites

Mattel Children’s Hospital at the University of California, Los Angeles (Los Angeles, CA) (Data Coordinating Center)
Arnold Palmer Hospital for Children (Orlando, FL)
Benioff Children’s Hospital at the University of California, San Francisco (San Francisco, CA)
British Columbia Children’s Hospital (Vancouver, BC, Canada)
Children’s Healthcare of Atlanta (Atlanta, GA)
Children’s Hospital at Montefiore (Bronx, NY)
Children’s Hospital of Philadelphia (Philadelphia, PA)
Children’s Medical Center (Dallas, TX)
Children’s Mercy Kansas City (Kansas City, MO)
Cincinnati Children’s Hospital (Cincinnati, OH)
St. Christopher’s Hospital for Children (Philadelphia, PA)
Texas Children’s Hospital (Houston, TX)

**Fig. 2:**
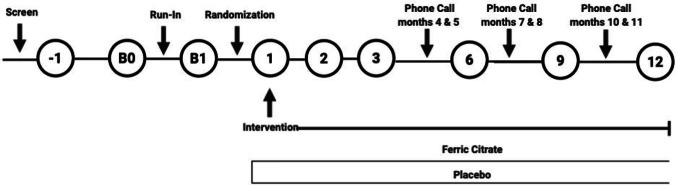
The Ferric Citrate and Chronic Kidney Disease in Children (FIT4KiD) trial schema. The FIT4KiD study is a randomized, double-blind, placebo-controlled, 12-month trial of 160 pediatric patients with CKD stages 3–4. It will test the hypothesis that, compared to placebo, ferric citrate will safely lower intact FGF23 levels. The study design includes a screening visit, baseline assessments, and post-randomization scheduled follow-up. Further details are provided in Table S1

The primary study hypothesis of the FIT4KiD trial is that compared to placebo, active treatment with ferric citrate will lower serum iFGF23 concentrations, measured over time (Table 3). To test this hypothesis, the FIT4KiD trial will recruit 160 pediatric participants between the ages of 6 and 17 years with CKD stages 3–4 (Table 4). After providing informed consent, study participants will be randomized to treatment with a weight-based dose of ferric citrate or treatment with placebo and will be followed for 12 months (Fig. 2). Participants weighing less than 31 kg will receive 3 g/day of ferric citrate; participants weighing greater than 31 kg but less than 51 kg will receive 5 g/day of ferric citrate; and participants weighing 51 kg or more will receive 6 g/day of ferric citrate. The total daily dosage will be divided into three doses to be taken with meals. Weight-based dosing was chosen instead of age-based dosing given that children with CKD are frequently small for age. Additionally, since dietary phosphate intake correlates with dietary caloric intake, and the latter correlates with body weight, weight-based dosing will best approximate dietary phosphate intake.

**Table 3:** Ferric Citrate and Chronic Kidney Disease in Children (FIT4KiD) study hypotheses. *FC*, ferric citrate; *FGF23*, fibroblast growth factor 23

Primary endpoint
Compared to placebo, from baseline, active treatment with FC will lower serum intact FGF23 concentrations
**Safety and tolerability endpoint**
Compared to placebo, active treatment with FC will be safe and tolerable
**Secondary endpoints**
Compared to placebo, from baseline, active therapy with FC will be associated with: -Increased hemoglobin -Increased serum transferrin saturation -Increased serum ferritin -Increased serum 1,25-dihydroxyvitamin D -Decreased serum parathyroid hormone
**Exploratory endpoints**
Compared to placebo, from baseline, active therapy with FC will be associated with: -Smaller decrease in estimated glomerular filtration rate -Decreased bone FGF23 -Increased osteoid thickness -Decreased biomarkers of bone turnover -Decreased phosphaturia -Increased serum calcium -Increased serum klotho -Decreased serum C-terminal (total) FGF23

**Table 4:** Participant inclusion and exclusion criteria

Inclusion criteria
Ages 6 to 17 years (inclusive)
Estimated glomerular filtration rate of 15–59 ml/min/1.73 m^2^ by the updated CKiD formula [60]
Serum phosphate within age-appropriate normal ranges
Serum ferritin < 500 ng/ml and transferrin saturation < 50%
For those patients treated with nutritional vitamin D, calcitriol, iron, and/or erythropoiesis-stimulating agents, doses must be stable for at least 2 weeks prior to screening
Able to swallow tablets
Able to eat at least two meals a day
In the opinion of the investigator, willing and able to follow the study treatment regimen and comply with the site investigator’s recommendations
**Exclusion criteria**
Current treatment with phosphate binders
History of allergic reactions, defined as rashes or hives, to ferric citrate or iron preparations
Current intestinal malabsorption, documented in the medical record
Anticipated initiation of dialysis or kidney transplantation within 6 months
Current or planned future systemic immunosuppressive therapy
Prior solid organ transplantation
Receipt of bone marrow transplant within 2 years of screening
Current pregnancy, current lactation, or female subjects who have reached puberty, unless using highly effective contraception
Patients participating in other interventional study (observational study participation is permitted)
Poor adherence to medical treatments in the opinion of the investigator

The primary analyses for this trial will evaluate changes from baseline in iFGF23 concentrations over 12 months between the treatment arm and the placebo arm. The analysis will use a linear mixed-effects model, with random participant effects accounting for repeated measurements, and random site effects accounting for clustering of participants into study sites. The model will include terms for treatment, time, treatment by time interaction (primary term for the inference in this study), baseline iFGF23, and stratification factors (study site, CKD stage, and urine protein/creatinine ratio) as covariates. Time will be modeled as two periods, months 1–3 and months 6–12, with the primary test based on the difference between treatment arms during the second time period [54]. A single interim analysis will be performed at 50% completion of 3 months of treatment or the first quarter of year 3 of the trial. The O’Brien/Fleming rules for interim analysis were used, assigning an alpha of 0.0054 to the midpoint analysis, and 0.0492 to the final analysis [59].

To evaluate the power of the trial, we carried out a simulation study using the analysis approach described above. Simulated data sets were created assuming that the mean level of natural log-transformed iFGF23 is the same at baseline in both arms, changes linearly during the first 3 months, is stable from 3 to 12 months in the treatment arm, and remains constant over time in the placebo group, as previously reported [54]. We utilized data from the CKiD cohort to estimate the design parameters (baseline mean, between sites and within and between subjects standard deviations) for the simulation. The simulation also assumed that 20% of participants would be lost to follow-up by month 12. The results of our simulation study showed that a sample size of 160 participants provides 80% power to detect a treatment difference of 26% between treatment vs. placebo.

Given that ferric citrate has been associated with improved hemoglobin concentrations [54–56], reductions in serum parathyroid hormone [53], and improved kidney outcomes [56], secondary objectives of this study include determining the effects of ferric citrate on anemia, indices of bone and mineral metabolism, and kidney function (Table 3). Serum creatinine will be measured over time, and GFR will be estimated using the updated CKiD formula [60].

We will also evaluate the safety and tolerability of ferric citrate, detailed in Table S1, including surveillance for gastrointestinal intolerance, iron overload, and hypophosphatemia. As described in Table S1 and Figures S1–S10, should gastrointestinal adverse effects, elevated transferrin saturation or ferritin, and/or hypophosphatemia occur, then study drug dosage will be adjusted. Regarding the possibility of citrate-induced enhanced intestinal aluminum absorption [61], in adult CKD studies, ferric citrate treatment was not associated with increased serum aluminum concentrations [17]. Nevertheless, as stated in the study protocol, administration of aluminum-containing compounds should be avoided in study participants.

Additionally, 24 UCLA participants will undergo iliac crest bone biopsy to determine the effect of ferric citrate therapy on the bone mineralization defect that is commonly seen in children with CKD [2]. Bone biopsy samples will also be assessed by immunohistochemistry for FGF23. A study by Pereira et al. demonstrated a correlation between bone FGF23 expression by immunohistochemistry and parameters of mineralization in pediatric and young adult patients with CKD stages 2–5 [23], underscoring the importance of evaluating bone FGF23 in association with histomorphometry. Bone biopsies will be performed only at UCLA, and there will be a separate consent form for the procedure. Patients will remain eligible for study entry even if they decline participation in the bone biopsy sub-study.

Participants will be withdrawn from the study if they experience one of the following: confirmed transferrin saturation > 70%, progression to kidney failure, initiation of phosphate binders, initiation of immunosuppression, desire of the patient or family to discontinue participation in the trial, loss to follow-up, or pregnancy. Medication adherence will be critical during the conduct of the trial. Therefore, the study will use a web-based, electronic medication adherence monitoring system called eCAP (Information Mediary Corporation, Ottawa, ON, Canada), which is a smart medication bottle cap that records real-time bottle cap removal times with an electronic timestamp. Additionally, dose reminders, pill counters, school diary of lunch-time administration, and self-reported adherence measures will be utilized throughout the study.

Possible anticipated limitations of the FIT4KiD trial relate to the use of FGF23 as the primary endpoint and medication adherence. Although it can be hypothesized that lowering FGF23 levels in patients with CKD may improve clinical outcomes, to date, there have been no trials directly targeting FGF23 to improve clinical outcomes in patients with CKD [62]. Therefore, it is still unknown whether or not FGF23 reduction would directly translate to improvement in hard clinical outcomes in CKD. Additionally, medication adherence may pose a challenge. In pediatric patients with CKD, rates of medication non-adherence are high; specifically, medication non-adherence for phosphate binders has been reported to be ~ 20–30% [63, 64]. In the recent COMBINE trial, which investigated the effects of lanthanum carbonate (a phosphate binder) and/or nicotinamide (an inhibitor of intestinal phosphate transport) on phosphate and FGF23 levels in adult patients with stage 3b/4 CKD, neither serum phosphate nor FGF23 significantly changed over time; however, study drug discontinuation was high, ranging from 25 to 42% across active treatment groups (compared to 14% in the placebo group), possibly limiting study conclusions [65].

In summary, the FIT4KiD trial will determine the impact of ferric citrate on FGF23 in pediatric participants with CKD stages 3–4. Given the associations of FGF23 with adverse clinical outcomes, interventions that safely lower FGF23 may improve long-term patient outcomes. Importantly, should ferric citrate safely and effectively reduce FGF23 concentrations, then the study findings may have important implications for the optimal management of mineral bone disorder in children with CKD.

## Supplementary information

## Supplementary Information

Below is the link to the electronic supplementary material.

Supplementary file1 (DOCX 264 KB)
