# Supplementary material for: A review of ferric citrate clinical studies, and the rationale and design of the Ferric Citrate and Chronic Kidney Disease in Children (FIT4KiD) trial
Source: Pediatr Nephrol. 2022 Mar 2;37(11):2547–57. doi: 10.1007/s00467-022-05492-7 (PMC9437144; doi:10.1007/s00467-022-05492-7)
Supplement: Supplementary file 1 — Supplementary file1 (DOCX 264 KB) [file 467_2022_5492_MOESM1_ESM.docx]

**A Review of Ferric Citrate Clinical Studies, and the Rationale and Design of the Ferric Citrate and Chronic Kidney Disease in Children (FIT4KiD) Trial**

**Supplementary Material:**

| **Table S1: Study Procedures**  **and Laboratory Tests** | **S** | **B0** | **B1** | **1M** | **2M** | **3M** | **4M** | **5M** | **6M** | **7M** | **8M** | **9M** | **10M** | **11M** | **12M** |
| --- | --- | --- | --- | --- | --- | --- | --- | --- | --- | --- | --- | --- | --- | --- | --- |
| **Study Procedures:** |  | | | | | | | | | | | | | | |
| Inclusion & Exclusion Criteria | X |  |  |  |  |  |  |  |  |  |  |  |  |  |  |
| Demographics | X |  |  |  |  |  |  |  |  |  |  |  |  |  |  |
| Medical History | X |  |  |  |  |  |  |  |  |  |  |  |  |  |  |
| Physical Exam |  |  | X |  |  |  |  |  |  |  |  |  |  |  | X |
| Vital Signs |  |  | X |  |  |  |  |  |  |  |  |  |  |  | X |
| Instruction on eCAP with  Distribution of eCAP |  | X |  |  |  |  |  |  |  |  |  |  |  |  |  |
| Adherence Reinforcement |  | X | X | X | X | X | X | X | X | X | X | X | X | X | X |
| Placebo/Drug Dispensing,  Return Pill Count,  Electronic Adherence Monitoring |  | X | X | X | X | X |  |  | X |  |  | X |  |  | X |
| Medical Adherence Measure |  |  | X |  |  |  |  |  | X |  |  |  |  |  | X |
| GI Questionnaire |  | X | X | X | X | X |  |  | X |  |  | X |  |  | X |
| Follow-up Telephone Visit |  |  |  |  |  |  | X | X |  | X | X |  | X | X |  |
| Adverse Event Ascertainment |  | X | X | X | X | X | X | X | X | X | X | X | X | X | X |
| Concomitant Medications | X | X | X | X | X | X | X | X | X | X | X | X | X | X | X |
| **Laboratory Tests:** |  | | | | | | | | | | | | | | |
| Safety Labs: CBC, serum creatinine, serum phosphate, TSAT, ferritin | X | X |  | X | X | X |  |  | X |  |  | X |  |  | X |
| Random urine for  protein and creatinine | X |  |  |  |  |  |  |  |  |  |  |  |  |  |  |
| Random urine for  creatinine, phosphate, & albumin |  | X |  |  |  | X |  |  |  |  |  |  |  |  | X |
| FGF23 | X | X |  | X | X | X |  |  | X |  |  | X |  |  | X |
| Blood samples for storage for  bone markers and future use | X | X |  | X | X | X |  |  | X |  |  | X |  |  | X |
| LithoLink 24-hour urine collection |  |  | X |  |  | X |  |  |  |  |  |  |  |  | X |
| Random urine for storage for markers of kidney disease and future use |  | X |  |  |  | X |  |  | X |  |  |  |  |  | X |
| Urine for pregnancy (if applicable) |  |  | X |  |  |  |  |  | X |  |  |  |  |  | X |

S = Screening Visit; B0 = Initial Baseline Visit; B1 = Final Baseline Visit; 1M = First Month; 2M = Second Month; 3M = Third Month, etc.

**Dose Adjustments, Modifications, and Delays**

Dose adjustment will take place if and when one of the expected adverse events occur. The full Manual of Operations contains additional details regarding procedures for study drug dose adjustments or discontinuations for participants who eat fewer than 3 meals a day, who are hospitalized, or those who develop inter-current illness or other side effects.

**Gastrointestinal Side Effects**

The PRO-CTCAE™ Measurement System will be used to assess the following gastrointestinal symptoms: nausea and vomiting, abdominal pain, constipation, diarrhea, and gas and bloating. This tool is specifically developed to capture symptomatic adverse events among children by self-report (or in the case of children younger than 7 by caregiver report). If a participant develops gastrointestinal side effects that he or she considers to be intolerable, or that are considered intolerable by the site investigators, dose adjustment will be at the discretion of the site investigator. Other appropriate actions may include reduction of the dose of study drug or short drug holidays, as described in the Manual of Operations.

**Monitoring of Iron Status and Serum Phosphate Concentrations**

Safety monitoring will include measurement of serum transferrin saturation (TSAT), ferritin, and phosphate. If elevated TSAT, elevated ferritin, and/or hypophosphatemia is detected, then the following flow charts will be followed (Figures S1-S10).

As variability in iron parameters is common, elevations in TSAT or ferritin will first prompt repeat testing, without stopping study drug. Also, as iron parameters can be affected by the timing of oral iron intake, participants will be asked to hold their study medication, and ferrous sulfate, on the days of blood testing.

Because serum phosphate levels are tightly regulated, and reductions in dietary phosphate absorption are accompanied by reductions in urinary phosphate excretion, reductions in serum phosphate levels with enteral phosphate binders are unlikely. Mild reductions in serum phosphate levels are not accompanied by symptoms; are often transient; and may be due to diurnal variation in serum phosphate, post-prandial status, and/or laboratory processing. Therefore, mild hypophosphatemia will first prompt repeat testing, without stopping study drug. However, if severe hypophosphatemia (defined as serum phosphate < 1.6 mg/dl) is detected, then the study drug will be held immediately until the results of repeat testing are known and assessed.

**Figure S1: Elevated Transferrin Saturation Flow Chart**

**Figure S2: Elevated Ferritin Flow Chart**

**Figure S3: Mild Hypophosphatemia (First Episode) Flow Chart**

**Figure S4: Mild Hypophosphatemia (Second Episode) Flow Chart**

**Figure S5: Mild Hypophosphatemia (Third Episode) Flow Chart**

**Figure S6: Severe Hypophosphatemia (First Episode) Flow Chart**

**Figure S7: Development of Mild Hypophosphatemia after Having One Confirmed Episode of Severe Hypophosphatemia Flow Chart**

**Figure S8: Development of Severe Hypophosphatemia after Having One Confirmed Episode of Severe Hypophosphatemia Flow Chart**

**Figure S9: Development of Mild Hypophosphatemia after Having One Confirmed Episode of Severe Hypophosphatemia, Followed by a Confirmed Episode of Mild Hypophosphatemia Flow Chart**

**Figure S10: Development of Severe Hypophosphatemia after Having Two Confirmed Episodes of Severe Hypophosphatemia Flow Chart**
